# A systematic review on machine learning models for online learning and examination systems

**DOI:** 10.7717/peerj-cs.986

**Published:** 2022-05-18

**Authors:** Sanaa Kaddoura, Daniela Elena Popescu, Jude D. Hemanth

**Affiliations:** 1 College of Technological Innovation, Zayed University, Abu Dhabi, United Arab Emirates; 2 Faculty of Electrical Engineering and Information Technology, University of Oradea, Oradea, Romania; 3 Electronics and Communication Engineering, Karunya Institute of Technology and Sciences, Coimbatore, Tamil Nadu, India

**Keywords:** Machine learning, Online learning, Online examinations, Authentication, Fraud detection, Security

## Abstract

Examinations or assessments play a vital role in every student’s life; they determine their future and career paths. The COVID pandemic has left adverse impacts in all areas, including the academic field. The regularized classroom learning and face-to-face real-time examinations were not feasible to avoid widespread infection and ensure safety. During these desperate times, technological advancements stepped in to aid students in continuing their education without any academic breaks. Machine learning is a key to this digital transformation of schools or colleges from real-time to online mode. Online learning and examination during lockdown were made possible by Machine learning methods. In this article, a systematic review of the role of Machine learning in Lockdown Exam Management Systems was conducted by evaluating 135 studies over the last five years. The significance of Machine learning in the entire exam cycle from pre-exam preparation, conduction of examination, and evaluation were studied and discussed. The unsupervised or supervised Machine learning algorithms were identified and categorized in each process. The primary aspects of examinations, such as authentication, scheduling, proctoring, and cheat or fraud detection, are investigated in detail with Machine learning perspectives. The main attributes, such as prediction of at-risk students, adaptive learning, and monitoring of students, are integrated for more understanding of the role of machine learning in exam preparation, followed by its management of the post-examination process. Finally, this review concludes with issues and challenges that machine learning imposes on the examination system, and these issues are discussed with solutions.

## Indroduction

Machine learning (ML) is a pioneering area of research in artificial intelligence. ML has a broad application area, from running search engines to protecting passwords. ML is the study of machines learning from human behavior to solve simple to complex problems (Lv & Tang, 2011). Machine learning algorithms revolutionize real-world applications and research directions. Machine learning makes systems more intelligent and automated to handle high-dimensional and complex data (Sarker, 2021). ML is adept at knowledge acquisition, learning, predicting, and solving problems. In real-time, ML saves the time and efforts of every human. It has become inevitable for every human to make their everyday lives smoother from multiple perspectives. This article discusses the role of ML in the academic area. During the COVID pandemic crisis, the agony of students and the hardships faced by education are many. The offline classes were not possible due to widespread virus threats (Tarik, Aissa & Yousef, 2021). The protection of students’ health and safety was a priority. The educational institutions are forced to cancel physical learning sessions in a classroom environment, and conducting examinations is challenging. E-learning, remote learning, and online education became a solution as it’s the only way out for continuous learning.

The examinations or assessments were considered necessary to be conducted online mode. The online education mode came to rescue the aid of students in completing their courses without a break in academia. The exam management system is the most affected in academia during the lockdown scenario. ML played a significant role in paving students toward their academic completion with examinations. The student data are a prerequisite for any analysis related to the academic perspective. The data mining of the student’s data of respective classes and research helps the educational infrastructure to develop a support system for the students and teachers in preparation for final assessments.

The student learning management data contribute to designing a decision-making model for prediction or analysis in the educational domain (Lavoué et al., 2017). Machine learning approaches are the key to the development and process of these learning and assessment models. However, the sample student data should be adequate and informational. The ML models are beneficial in the prediction analysis to study student performance based on the data. This futuristic prediction characteristic of ML facilitates improving student performance and provides early intervention in learning and achieving success in examinations (Sandra, Lumbangaol & Matsuo, 2021). The main advantage of the ML model is its capacity for accurate prediction with limited student data resources. The contribution of ML models proved to be substantial in student academic performance predictions, with learning outcomes aiding in preparation for upcoming examinations (Balaji et al., 2021). The unique ability to explain and interpret enables easy adaptation and operation for students, teachers, and administration. ML requires basic skills and primitive knowledge for its operation that can be implemented in education (Linardatos, Papastefanopoulos & Kotsiantis, 2020).

There is clear evidence that ML is a paradigm shift in the education field to be innovative, interactive, and personalized for collaborative learning. Whether online or offline learning mode, ML has proved to be valuable in outcome-based education for professional or career perspectives of students. The teaching strategy has been shifted to outcome-based education; the academic curriculum has been redefined based on program and course outcomes. For this outcome-based model, the prediction is the basis for building up the curriculum for student assessment preparation and evaluation. There is a demand for constant observation and frequent measurement of student learning ability before appearing examinations. The students’ examinations are centered on these curriculums and Program Educational Outcomes (Yanes et al., 2020). ML serves as an exoskeleton for the instructor to plan the instruction and evaluation models. ML provides teachers a platform for multifaceted presentation, simulation, and automated student feedback. ML bridges the gap between students and teachers for more interactive learning and proximal developments (August & Tsaima, 2021). This article is intended to discuss the impact of machine learning on an event of the learning process, “Examinations”. The key features of this review paper are the role of ML in the examination process during the lockdown and its emergence, multifaceted role in student assessment.

With the current onset of pandemic situation, educational institutions, industries, government organizations, *etc.* have shifted their inclination towards online mode of teaching/learning, communication and other interactions. In such scenario, there is a high probability of false-doing such as malpractices in examinations and other negative aspects. Hence, automated systems for online interactions must be well-equipped to overcome these drawbacks. The rationale behind this review is mainly focused on this concept by highlighting the merits and demerits of various ML models which can assist in the variety of online applications.

This review is specifically intended for educational institutions who carry out teaching and learning in the online mode. Proper selection of the ML based automated system will enhance the efficiency of online learning, online examinations, *etc.*. Government organizations which conduct nation-wide/state-wide online examinations also will benefit out of this review.

The motives of this work are:

• To summarize an overview of machine learning and the primary classification of its algorithms.
• To Identify research gap with research questions for ML in Exam Management with search objective.
• To discuss the need for Machine Learning in the Lockdown scenario.
• Analyzing the role of ML in different phases of examination is based on research questions.
• To address the issues and limitations of Machine Learning in the Exam Management System.

## Machine Learning: an Overview

**Figure 1:**
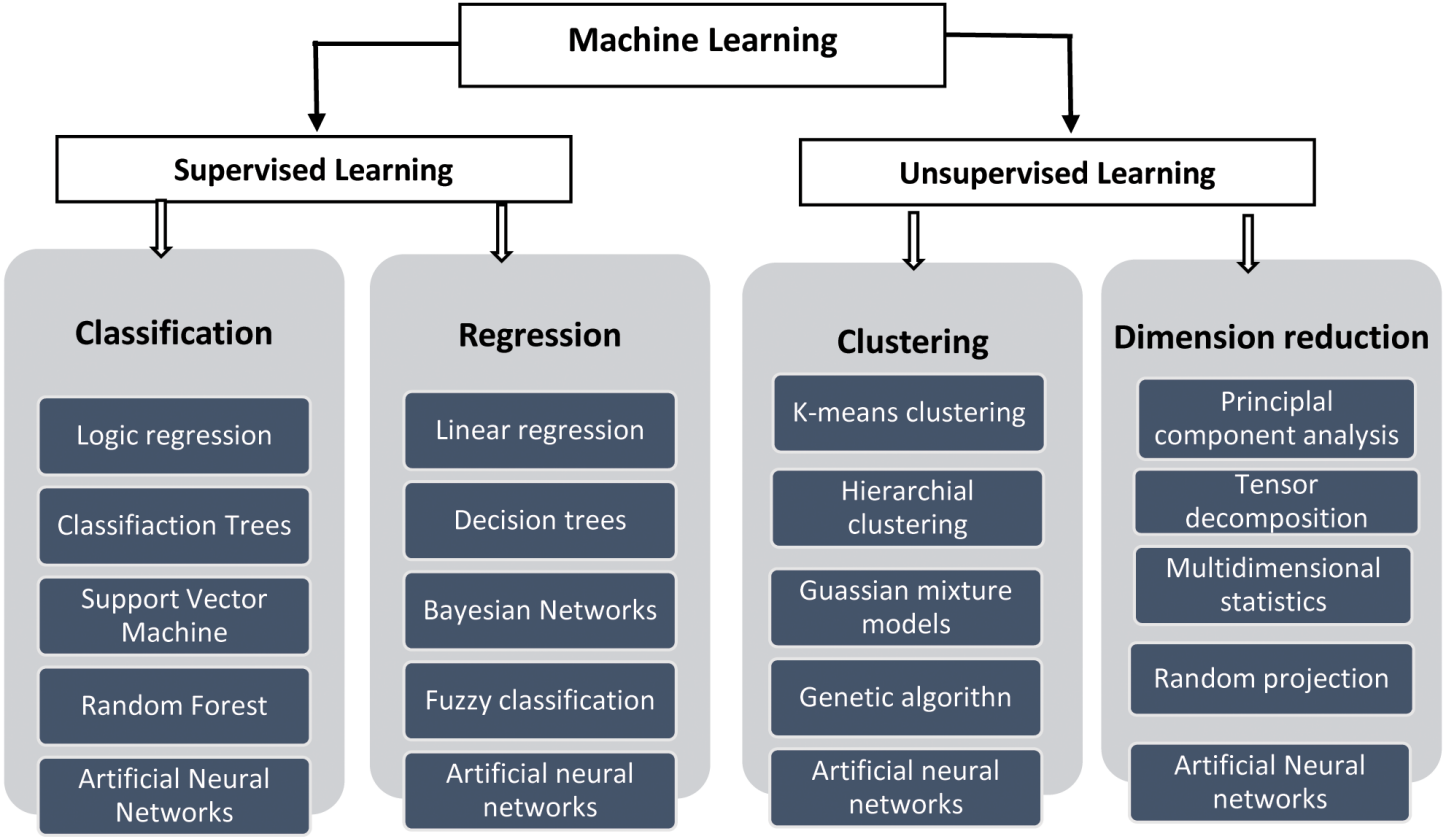
General classification of Machine Learning.

In artificial intelligence, Machine Learning is the study of how machines can learn human behavior and imitate it for decision making and solving complex problems. The machine learning algorithms started to evolve in 1970 though the term was coined in 1959. The main classification is based on their learning and testing the validity of the proposed model behavior as to whether supervised or unsupervised (Louridas & Ebert, 2016). In supervised learning, classification and regression algorithms utilize training data for prediction. The prediction is validated by the test set data. On the contrary, the machine must find its solutions using the training data in unsupervised learning. The general machine learning approaches and their classification with their techniques are displayed below in Fig. 1.

## Search Strategy and Research Questions

### Identification of research gap

Machine Learning is a vast area with multiple applications in different fields. This review is based on its application in the field of academia. When the generalized survey on ML in education was performed, several papers exhibited the role of ML in learning and the prediction of student performance. However, there was limited focal point review on the extensive role of ML in exam management. Systematic exam management is the need of the hour under lockdown situations. The ML is pivotal in automation and efficient in monitoring and regulation of a sequential examination process. If ML is not implemented the examination process, it may tend to become complicated with huge student data analysis. The manual timetable process can be challenging and may consume more human efforts and time. This inadequacy of work on ML in exam management systems motivated the authors to revisit the papers for the search strategy. This paper tracks the importance of ML from beginning to end of the examinations as an entire cycle. This review paper is structured as a sequence of activities aiming for a complete understanding of the pivotal role of ML in Exam Management Systems, from preparation to assessment and grading.

### Search strategy

**Figure 2:**
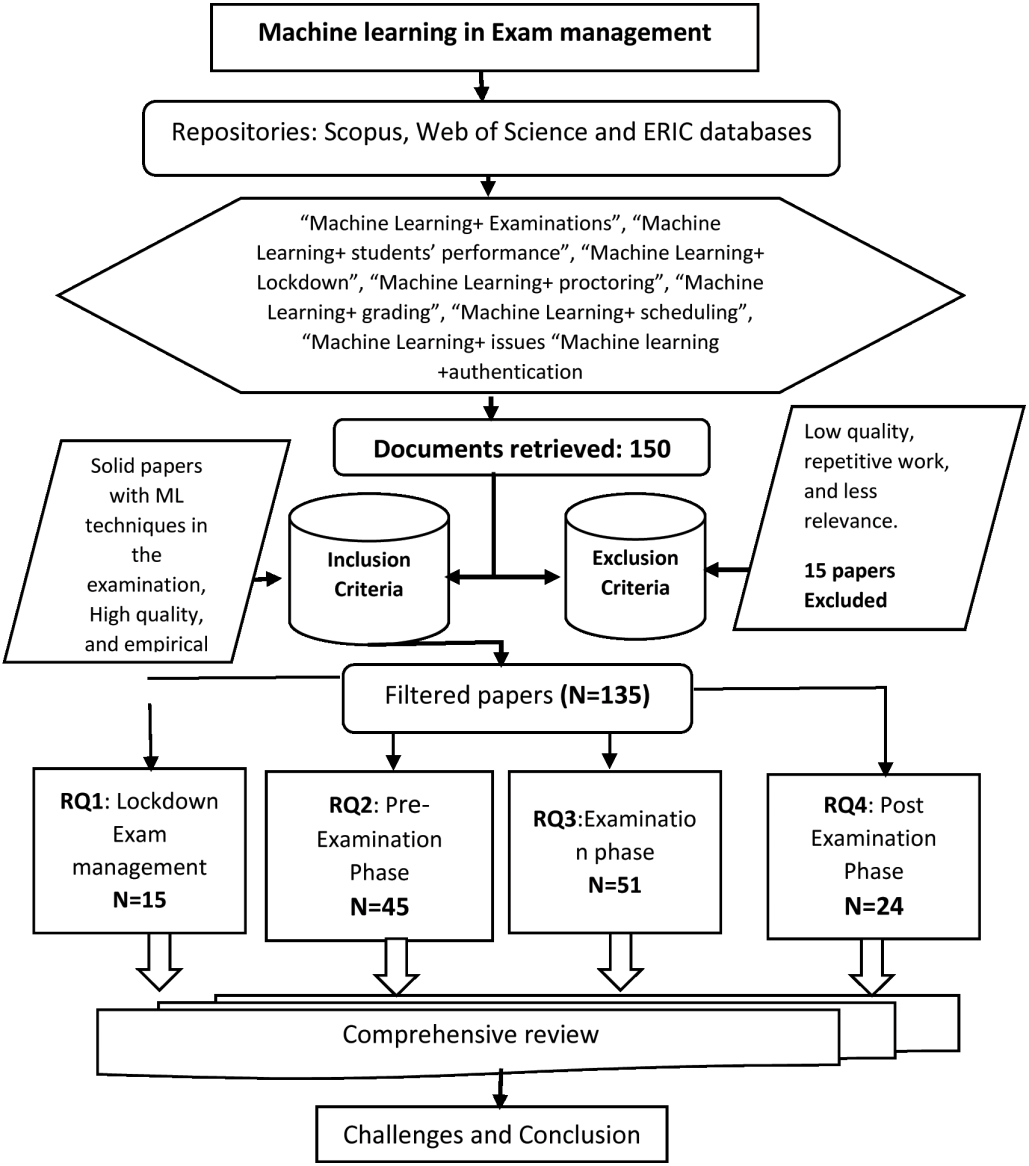
Search strategy for ML in the exam management system.

The motivation of this work is to elucidate empirical evidence supporting the effectiveness of machine learning on online preparation, assessment, and evaluation of student performance. For this review, an extensive search on machine learning was performed. The papers are selected based on recognized and accredited publications such as IEEE, Springer, Elsevier, Wiley, ACM, and International Journals. The articles are indexed in Scopus or web of sciences available in research databases. Machine learning is a popular topic in exhibits and conferences. The recent conference papers with many citations and novel ideas are also included in this study. The specific documents with machine learning keywords are retrieved, ensuring practical relevance in selecting each article. About 140 papers were selected from the last five years (some exceptional previous papers were also included). The search strategy is displayed below in Fig. 2.

### Research question formulation

The following research questions are based on the segmentation of the LEMS:

• RQ1: What is the significance of ML in education and examination during this lockdown scenario?
• RQ2: How does ML play a role in preparing students for examination through prediction?
• RQ3: What is the role of ML during examination in authentication, scheduling, and proctoring students?
• RQ4: How is ML beneficial in the post-exam process like grading and feedback?

**Figure 3:**
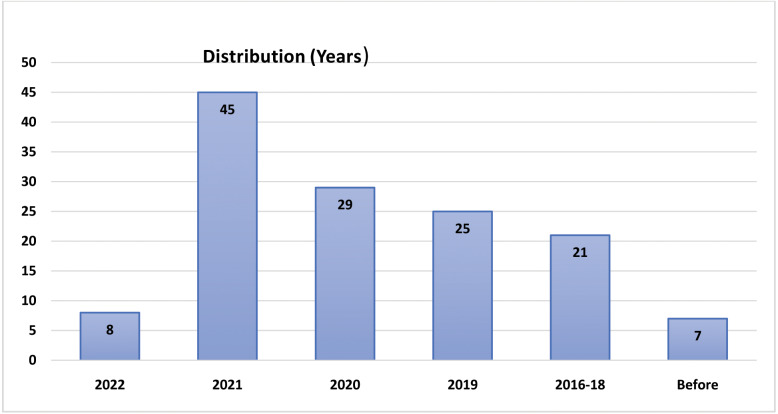
Distribution based on years.

**Figure 4:**
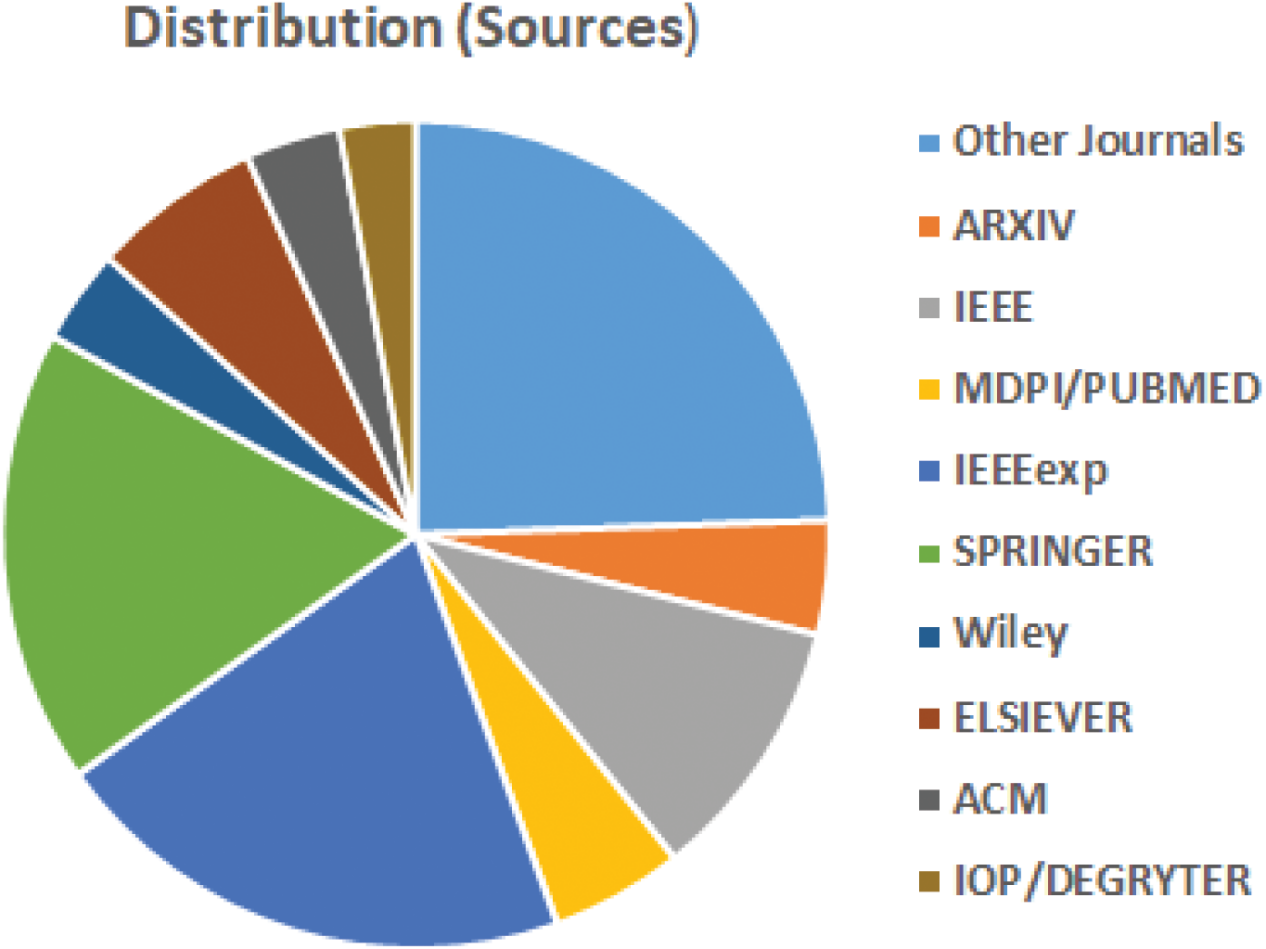
Distribution based on sources.

The above questions are formulated, and this leads into the following divisions: the significance of ML in current lockdown, Pre-Exam phase or preparation phase, Examination phase, Post examination phase (Evaluation and grading), and issues and challenges are discussed, followed by a conclusion. The distribution of retrieved papers based on years and sources are given in Figs. 3 and 4. For LEMS, most of the articles are chosen between 2019–2022, which has a bearing on the lockdown era.

## The Emergence of ML in Lockdown Exam Management Systems

There is an unmistakable emergence of the use of Machine Learning tools in the education sector during pandemic times. The contemporary research supports that ML has been a powerful tool implied in learning and assessment during these pandemic times. There is a profound shift in behavior for every student from learning in the classroom to online mode. The pandemic crisis has increased students’ emotional sensitivity, they are in anguish, and there is a big question mark about their future careers (Akour et al., 2021). In several countries, the ministry of education has suspended regular school or college. Face-to-face studies and group education were barred as a precaution. Distance education mode and assessment became obligatory at all levels, from kindergarten school to a doctorate or scholar defense exam (Tarik, Aissa & Yousef, 2021). The personal face-to-face contact to identify the student’s attentiveness and make interventions on the spot is dispensed. The performance prediction can be synchronous; the academic interventions online necessitate monitoring the asynchronous activity of the individual student.

The Machine Learning methods are experts in monitoring. It is also used to develop models to predict students at risk in advance to get the same benefit as in real-time (Karalar, Kapucu & Gürüler, 2021). The ML lends its hand the extra mile to the teachers for smart attendance, teaching, and conducting assessments. The ML made virtual classrooms and assessments possible; the survey was conducted with students for determining student satisfaction during emergency remote learning using Machine Learning. The results indicated that the students favor remote education and assessments (Ho, Cheong & Weldon, 2021). Academic growth is highly impacted due to COVID. The COVID brought a severe setback and sudden change in the modus operandi of academic structure. There are consequences due to COVID, affecting students’ academic standards and behavior to embrace new changes with fear of the future. The ML has been a silent hero that helped the field of academics and assessment to continue with the same integrity (Agarwal et al., 2021). The ML lays building blocks for students to have a safer world and stay ahead amid setbacks.

### The examination cycle in the lockdown exam management system (LEMS) has four significant steps

1. Pre-Examination Phase (Preparation)
2. Examination Phase (Conduction of Assessments)
3. Post examination phase (Grading with Integrity)
4. Resolving Issues with ML implementation in Examinations

**Figure 5:**
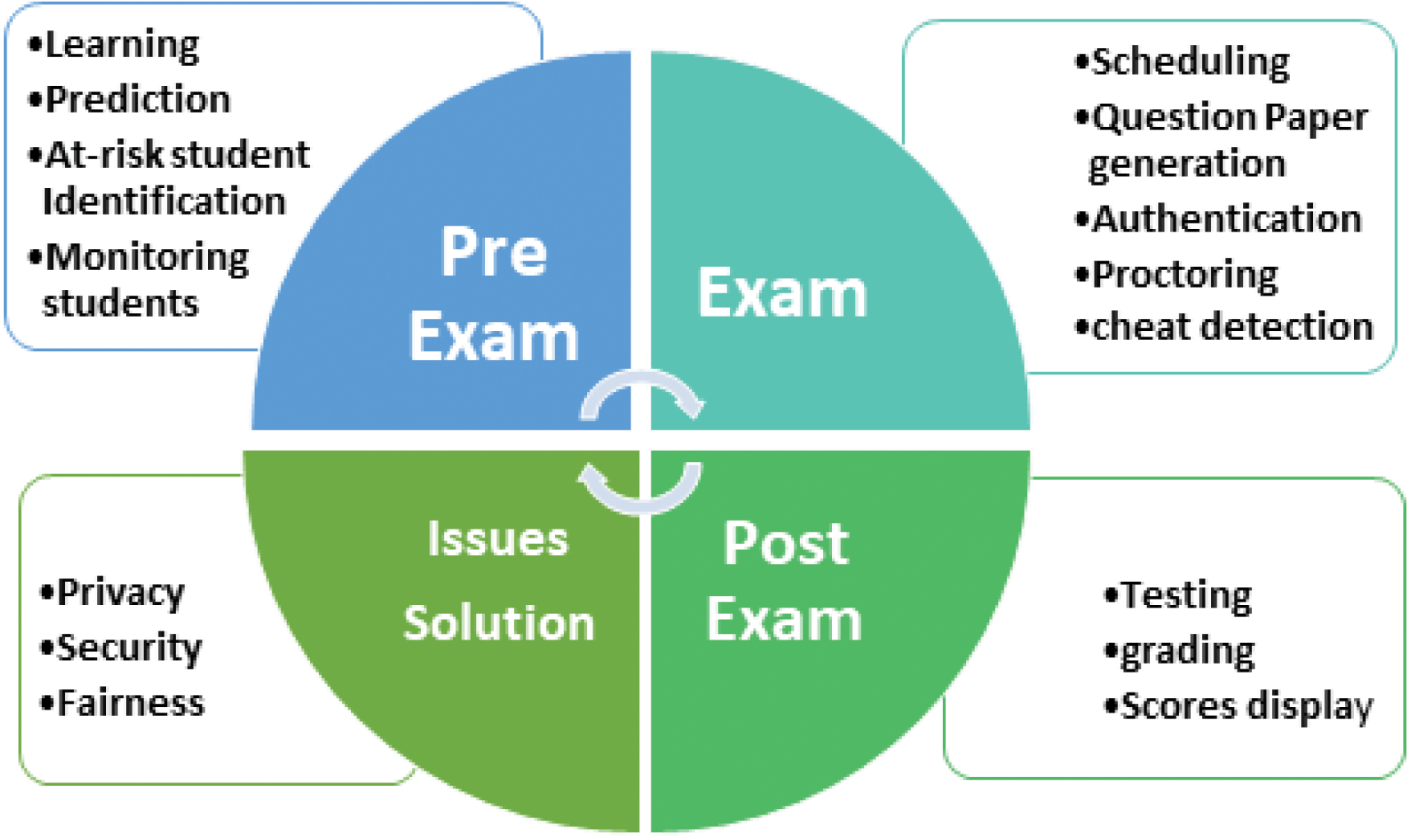
The exam cycle in LEMS.

The exam cycle and its subareas focused on this article are illustrated in Fig. 5.

## Role of ML in Pre-exams (Preparation Phase)

### Adaptive learning before examination

Adaptive learning is an approach where the study materials, teaching methods, and inculcation are tuned with the proactive curriculum. The individual learning path is designed for the maximum enrichment of the student’s knowledge and thus enhances the student’s academic achievement. ML must identify the students who are low-level performers to pay personal attention. The ML boosts the student to overcome his vulnerability in the learning process through tailor-made learning modes. However, each student has their behaviors in learning attitude and continual monitoring (Embarak, 2021). ML has a role in designing web-based learning systems by an interface. The learning interface must be adaptive and satisfy individual learner criteria for creating personalized learning. The downside is the non-availability of a collaborative learning approach that has interactions for more efficiency (Oboko et al., 2016). The concepts of brain dominance and students’ psychological behavior serve as input for ML-based models. There is a prerequisite of catering the personalized education to the individual student’s aspiration. This psychological behavior-based ML concept is proposed and tested in practical conditions (Oke et al., 2016).

Modern technological developments demand an intelligent learning environment for students. The teaching strategies should be adaptive to individual student learning characteristics. The ML models enlighten more on personalized adaptive learning for effective pedagogy. The unique student characteristics and personal development are its prime criteria in designing education-based ML models (Peng, Ma & Spector, 2019). Recently, AI-based learning has been prominent in implementing adaptive learning. The systematic mapping method of AI is beneficial for education purposes. The review clearly shows that ML with AI dawns a new era for adaptive learning, helping students in knowledge enhancement (Kabudi, Pappas & Olsen, 2021). The research revisits challenges and issues with the widespread implementation of adaptive learning. The frequent redesigning of curriculum, loss of control in the hands of the faculties, and reluctance to cope with the learning of new tools for updating technology are the issues to be addressed (Mirata et al., 2020).

### Predictive analysis of student performance

Assessing a student’s performance at strategic time intervals before exams gives an impetus for improved teaching methods that assist students in achieving success in their exams. The various ML methods and algorithms are extensively used to predict performance in many student communities. The details are inferred below in Table 1.

**Table 1:** Role of ML in predictive analysis.

**Paper ID**	**Student dataset**	**ML technique used or compared**	**Inferences**
Kotsiantis, Pierrakeas & Pintelas (2004)	University students for distance learning	(Naïve Bayes) NB, 3-NN, RIPPER	Early predictions are accurate using NB
Koutina & Kermanidis (2011)	Postgraduate students	NB, 1-NN, Random Forest (RF), and SMO	The combination of NB and 1-NN is efficient with sampling
Sweeney et al. (2016)	Public University	Factorization Machines (FMs), Random Forests (RFs), and the Personalized Linear Multiple Regression (PLMR)	A hybrid and efficient method of RF and FM is proposed.
Xu, Moon & Van der Schaar (2017)	Degree students	A novel method is proposed (latent factor model-based course clustering method)	A progressive prediction architecture is proposed for evolving performances.
Fagbola et al. (2019)	Programming courses	Linear Regression (LR), M5P decision tree	Designed and evaluated Mobile Interface with ML statistical models
Hussain et al. (2018)	Learning session data	LR, ANN, SVM, NBC, and DT	The SVM and ANN models are accurate
Sekeroglu, Dimililer & Tuncal (2019)	Secondary school	Backpropagation (BP), Support Vector Regression (SVR), and Long-Short Term Memory (LSTM),	SVR has the highest prediction rate accuracy.
Jain & Solanki (2019)	Multiclass students	DT, RF, Gradient Boost	RF outperforms for correlative analysis of student performance
Alshabandar et al. (2020)	Massive open online courses	Root Mean Square Error (RMSE) and R-squared	Pass or Fail analysis. But lack of inclusion of temporal features.
Shashi, Sunil & Kumkum (2020)	University students	e Logistics Regression, Naïve Bayes, K-Nearest Neighbor, Decision Tree (DT), Support Vector machine	DT outperforms the acquired dataset.
Dhilipan et al. (2021)	College students	Binomial logical regression, Decision tree, Entropy, and KNN	Binomial Logical regression is accurate.
Pallathadka et al. (2021)	UCI machinery student dataset	Naive Bayes, ID3, C4.5, and SVM	SVM has more accuracy and less error rate
Li & Liu (2021)	High school students	CNN, RNN, and DNN	DNN is accurate for a vast dataset
Alnassar et al. (2021)	Virtual learning environment	Support Vector Classifier (SVC), k-Nearest Neighbor (k-NN), Artificial Neural Network (ANN)	K-NN algorithm is more suitable for critical analysis.
Baashar et al. (2022)	Higher education	Various ANN algorithms	ANN with data mining methods is more prominently used in higher education
Albreiki, Zaki & Alashwal (2021)	Multiple datasets	SVM, DT, NB, KNN	There is a need for dynamic
Priya, Ankit & Divyansh (2021)	Secondary schools	Logistic regression, ANN, SVM	Logistic regression performs better in the prediction
Hussain & Khan (2021)	Secondary and intermediate levels	DT, GA based DT, KNN, GA found KNN	GA based DT outperforms all the other methods
Xiao, Ji & Hu (2021)	Survey on different students	DT, NB, MLP, RF, SVM, KNN	Shortcomings of each prediction method were discussed
Qiu et al. (2022)	E-learning environment	SVM, NB, DT	A behavior-based prediction model is designed and evaluated.

### Identification of at-risk students before examinations

**Figure 6:**
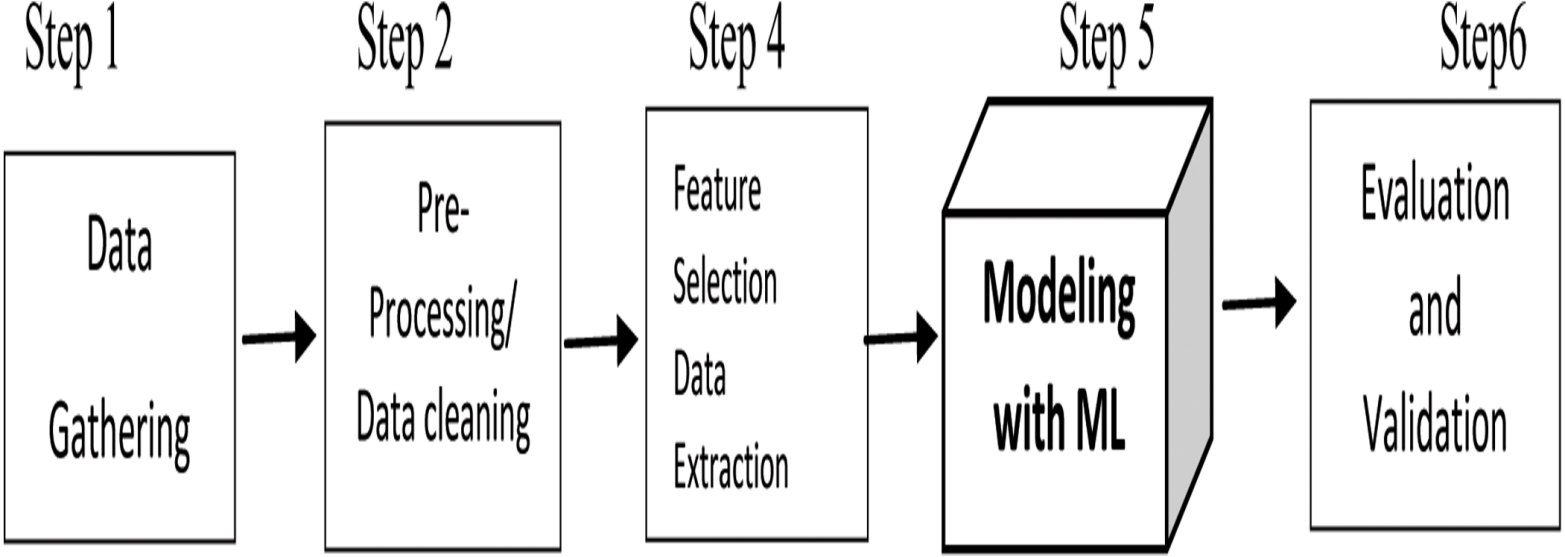
Steps for identification of at-risk students.

The ML has a significant role in predicting forthcoming performance. Besides that, it is also proficient in identifying vulnerable students (low achievers) and a risk category of failure in exams. This prediction and student identification aid the teachers in designing a practical pedagogy for developing personalized monitoring and individual attention. ML facilitates more students to achieve success in coming examinations. The generalized model to predict any at-risk students involves five mandatory steps, as shown in Fig. 6.

The student dataset is preprocessed in an at-risk model, and the required features are extracted. The features such as academic, demographic, social and behavioral factors of the students in prior semester are considered for data set creation as input into ML model for at-risk identification. The ML models are based on algorithms such as Naïve Bayes, Decision Trees, Random Forest, Neural Networks, and even hybrid methods (Marwaha & Singla, 2019). ML with data snapshot analysis helps find underperforming students who need more attention in programming classes. The students can have rehearsal tasks for more understanding of the concepts (Ahadi et al., 2015). The students’ data of periodic assignments submission helps the ML model predict the students who are prone to risk in the examination (Falkner & Falkner, 2012). When applied to the actual log of Learning Management Systems (LMS), the ML methods can determine up to forty percent accuracy in the early detection of at-risk students. The casual students’ behavior, such as their e-book reading habits with an appropriate ML model, can predict students’ at-risk using classifiers (Chen et al., 2021). By closely monitoring ML techniques output, the instructors can intervene with the student and offer additional support and more adaptive teaching (Kondo, Okubo & Hatanaka, 2017).

The ML also helps the university administration identify at-risk students before examinations. ML resolves the issues of rising university attrition. ML models applied with the administrative data give the university administration a predicting insight of students who may discontinue in the middle of the course and take necessary steps to prevent attrition (Berens et al., 2018). In a virtual learning environment of college education, the ML methods identify at-risk students and even marginal students before the examination. More success for these marginal students can be achieved by providing due attention to the examination (Chui et al., 2020).

In massive online open courses (MOOC), ML predictions are more efficient in identifying vulnerable students and improving these students’ performance with ML-based learning achievement mode (Al-Shabandar et al., 2019). Sometimes, manual methods fail to identify some demographic or financial issues that may cause students to be at-risk before examinations. ML methods are pragmatic and more potent in determining the student’s at-risk conditions, which manual processes overlook (Soobramoney & Singh, 2019). There are instances where the socio-economic situation leads a student towards academic desertion before completion. There has been a forty to seventy percent increase in at-risk students in countries like South America. The ML was applied to such scenarios for detecting students and focused on these students’ issues on a national scale (Zea, Reina & Molano, 2019).

The ML has proved its extreme impact on the student population, from university students to the K-12 classes, which determines a career path. The prediction promotes the scholastic achievement of such altering their career path (Li et al., 2020). The ML models can be implemented in levels for more accuracy in prediction. A model with three levels of classifiers can achieve eighty percent of accuracy in prediction (Latif, XianWen & Wang, 2021). The ML model’s prediction is based on static data, but they can even stack the students’ weekly performances and their growth in the academic pursuit and bring out a few at risk. It is a repeated measure process that makes it possible to identify the at-risk students throughout the academic year, not a one-time event (Koutcheme et al., 2022). Whether it is its online courses or any virtual learning environment, it is established that there is evidence of the inevitable role of ML, dedicated to the design of prediction models for at-risk students (Adnan et al., 2021). The ML analyzes precisely and competently, helping the students to receive support and proper mentoring from teachers or to avoid dropping out of examinations.

### Monitoring student attendance and learning

The examination may be a pivotal event in every student’s life; the preparation process is necessary. The student’s regular attendance in the virtual class and attentiveness is obligatory for preparation and appearance for examination. Most schools and universities regulate a mandated attendance percentage for appearing examinations. Machine learning can be implemented for face detection and face recognition for monitoring intelligent attendance systems. The low-cost ML method of the camera model is all ready to take photos of the students fifteen minutes before starting and ending the class, even in a real-time environment. This method is efficient than other ML models based on LBPH, Eigen face and Fisher face (Chowdhury et al., 2019). The convolutional neural networks (CNN) based ML method of smart attendance is ideal for online and virtual environments (Srivastava et al., 2019). Advanced ML methods also store multiple face data and can detect multiple faces in real time-consuming time. The principal component analysis (PCA) also takes the follow-up process of keeping the attendance information and redirects to the required email as an attendance sheet. The teachers or instructors should have a detailed summary of students’ attendance in their classes (Sawhney et al., 2019). The smart digital attendance applies CNN with a camera used by the students for more pixel clarity and pleasing light surroundings for detection and recognition (Devi & Narayanan, 2022). The ML supports facial expression detection for monitoring the physical presence of the students. The facial expression analysis gives information to teachers about the number of students present in their classes, not just physical attendance but also collective feedback on to what extent students could cope with the procedures (Ashok et al., 2020).

The ML has a substantial part in assisting smart attendance and an eye on learners’ attention. There is evidence of tracking the student’s attentiveness with ML-based multimodal biometric techniques, including eye gazing and behavioral biometrics involving body movements (Villa et al., 2020). In critical student training such as defense aircraft systems, ML was implemented with wearable sensors for eye tracking, cardiovascular, respiratory, and electrodermal classifiers to predict the accurate levels of learner’s involvement engagement in a training session (Carroll et al., 2020). In air-pilot classes, the machine vision-based ML approaches can be used to determine the students’ attentiveness. The refined identification classifiers such as eye gaze, head poses, and facial expressions can predict the student’s attention level (Goldberg et al., 2019). Any student who attends a class with physical attendance, attentiveness, and zeal, can successfully come up in the upcoming examinations.

## Role of ML in Exams (Examination Phase)

The core activities in carrying out the examination system are publishing the examination schedule, question paper generation, authentication of eligible students appearing for the examination, proctoring (supervision) of the examinations, and eliminating malpractices by appropriate steps.

### Scheduling examinations

**Figure 7:**
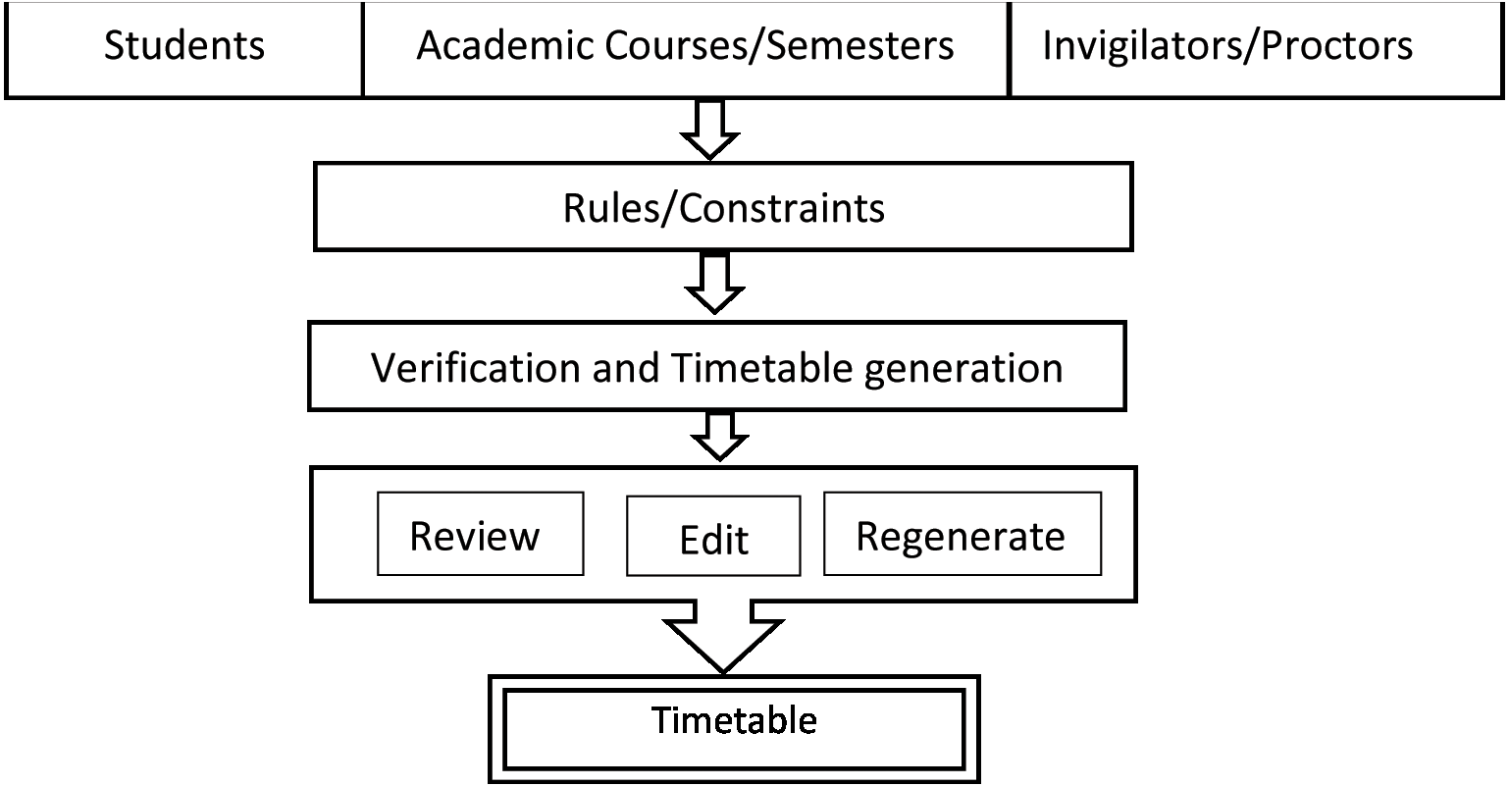
ML-based exam scheduler.

The publication of the examination timetable or schedule is the first step in conducting an examination. Machine learning has played a predominant role in scheduling since 1980. ML with artificial intelligence has proven to be capable of dynamic scheduling of examinations. The ML has already ruled the commercial industries in scheduling, execution, and automation. Now, ML is applied to education, payrolls, generating timetables, and conduction of examinations (Aytug et al., 1994). In universities, students are allowed to study extra credit courses parallelly, which may lead to overlapping in the examination timetable schedule. There will be difficulty in preparing timetables in these overlapping cases manually. The ML has a model that can automatically generate the timetable (Kumar et al., 2020). The ML-based examination schedule system architecture is given in Fig. 7.

The improved automation methods with iterative machine learning and optimization techniques will help conduct a fool-proof online or virtual examination. Neural networks and decision trees have a significant role in this ML-based model’s timetable generation by the iterative development method. ML for the examination process reduces the work burden of the instructors and invigilators to a large extent (Hutter, Kotthoff & Vanschoren, 2019).

### Question paper generation

Precaution: The students may use the internet to search exam questions and find answers in google or search engines during the examination. Based on this viewpoint, the questions can be changed with a different verbatim and paraphrased to discourage web searching (Golden & Kohlbeck, 2020). Another method is the generation of randomized multiple-choice questions using the ML method. These questions can form a part of the question paper of the online examination to reduce the repeated questions involving cooperative cheating (Tiong & Lee, 2021).

### Authentication of students

The verification of student identity is a pivotal step before the examination to avoid impersonation. ML methods can be implemented for the authentication of student identity. In some complex cases such as twins the multimodal authentication techniques are used for authentication of the individuals. The various existing methods of authentication are listed below in Table 2.

**Table 2:** The Role of ML in authentication.

**Paper ID**	**Year**	**Authentication**	**ML methods**
Rateria & Agarwal (2018)	2018	Offline signature	Hybrid classifier CNN and SVM
Gideon et al. (2018)	2018	Handwritten signature	Conventional neural networks
Bibi, Naz & Rehman (2019)	2019	Signature online/offline	ANN, NB, KNN, and SVM
Sadikan, Ramli & Fudzee (2019)	2019	Keystroke dynamics	ANN, KNN, and hybrid classifiers
Ryu, Yeom & Kim (2020)	2021	Multi biometric face/keystroke	Eigen Face and SVM
Geetha et al. (2021)	2021	Face recognition	Eigen Face and SVM
Deepika & Sneha (2021)	2021	Face detection	Logistic regression
Sukmandhani & Sutedja (2019)	2019	Face recognition	Self-organized Neural networks
Mehta (2019)	2019	Face and emotion	Local Binary Pattern Histogram
Kamencay et al. (2017)	2017	Face recognition	Convolutional Neural Networks
Adetoba et al. (2022)	2020	Face, video, and password	Logistic regression and SVM
Cerna et al. (2018)	2018	Face and fingerprint	Multitask Convolutional Neural Networks
RaviTeja, Brahmananda & Swasthika Jain (2020)	2020	Face and fingerprint	Convolutional neural networks
De Marsico, Petrosino & Ricciardi (2016)	2016	Iris recognition	ANN and SVM
Shelke & Bagal (2017)	2017	Iris identification	ANN and SVM
Traoré et al. (2017)	2017	Face, keyboard, and mouse	Exam shield application
Prabu, Lakshmanan & Mohammed (2019)	2019	Iris and hand geometry	Neural Networks and Bayes Networks
Joshy et al. (2018)	2018	Face, one time password, and fingerprint	Histogram of oriented gradients with KNN
Ryu et al. (2021)	2021	Continuous multimodal biometric	KNN, NB, and Random Forest
Labayen et al. (2021)	2021	Multimodal	Neural Networks and AI

### Proctoring (supervision) of examinations

The proctoring process of examinations requires continual monitoring and verification of student identity. The student’s face is initially registered before the commencement of the examination in a database. For continuous validation of a student’s face, an ML-based Convolutional Neural Network (CNN) collects images for face recognition and efficiently verifies the student’s identity (Asep & Bandung, 2019). The ML algorithms capture the face of the student during the examination and match it with the collected images for face recognition to confirm student attendance. This method is automatic and continuous during online proctoring (Ghizlane, Hicham & Reda, 2019).

The ML-based Proctoring software PROCTORU is widely used in online examinations. The ML can record numerous behavioral changes in a student. The head, hand, and eyes movements are tracked and are created as data points with webcam scanning. Suppose the symptom of mismatch pattern is detected, then immediately sent to the knowledge of the admin to keep close watch the abnormal student behavior safeguarding the security and integrity of the examination (Slusky, 2020). The ML with computer vision is ideal for face recognition and gesture detection in image processing. Real-time face recognition is possible by ML in OPENCV proctoring tool. The unsupervised examination is feasible due to ML (Pandey et al., 2020). The PROCTORIO tool with ML is used for live detection of human faces with automation. ML also supports the ID verification process during proctoring examinations. The 360-degree security camera was proposed in online proctoring for more coverage of the surrounding of the student. The sound is recorded, and movements are tracked during an examination. The webcam and security camera can monitor the surrounding of the students with ML algorithms (Turani, Alkhateeb & Alsewari, 2020). This method is advantageous for both live proctoring and recorded proctoring.

The artificial neural networks with ML pave the way for data analytics in the authentication phase of invigilation. The unique ability of ML is to retrieve the required data and label them from the unstructured data is used in recognitions and detections for proctoring tools (Bhardwaj, 2020). In remote online proctoring systems, ML verifies the user and tracks the student’s gazes. When integrated with RESPONDUS, it can lock tabs to prevent cheating by locking down the browser (Vamsi & Ashwin, 2021). Integrated remote supervision with machine learning was proposed in IRS-MLA. This model enables a hybrid learning and examination management system for the student’s maximum benefit, providing a safe environment with remote supervision (Lu, Vivekananda & Shanthini, 2022). The supervision with ML proved to be more efficient and safer during the pandemic times. The online ML-based English hierarchical test was tested against the traditional test system. The comparative analysis showed that ML proved efficient in maintaining fairness and speed (Wang et al., 2021). The evidence suggests ML and ML-based proctoring is influential and trustworthy in all the revisited cases. Emerging AI technologies impact the future of exam management in a far better way (Vincent-Lancrin & Van der Vlies, 2020).

### Fraud or cheat detection

The advancements in technology promote remote proctoring and unsupervised methods; there is a susceptibility to cheating and fraud during the examination. Cheating in the online assessment is termed ”Academic Dishonesty”. The cheat detection can be done in-situ or in a-posteriori detection. In live proctoring, the detection should be in-situ, but in many cases, a posteriori detection is done with the help of ANN and SVM (Küppers et al., 2022).

**Figure 8:**
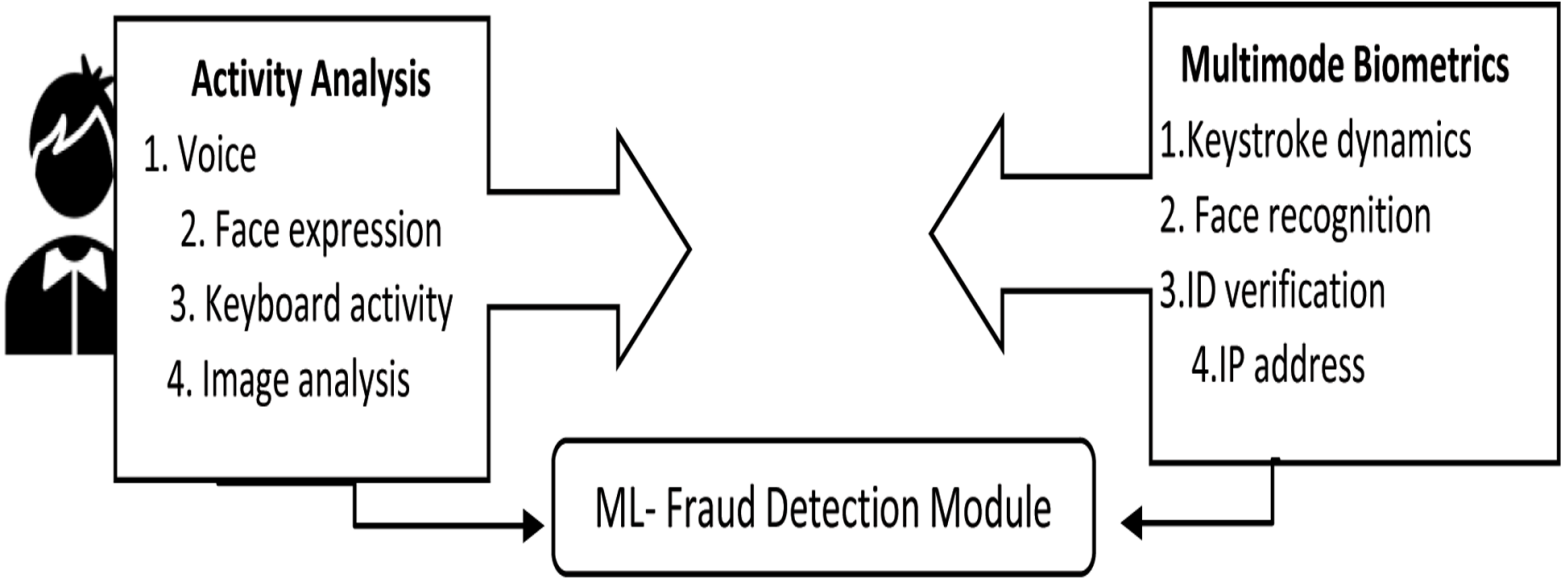
ML-based fraud detection module.

(a) ***Plagiarism:*** Students tend to plagiarize the content during examinations. The comparison of text structure can be done by statistical approaches. Finger printing and term frequency matrices (TFM) are used to detect the column of words with the frequency of words in rows of the matrices. This method is effective with the downside of consumption of time, The second method to detect plagiarism implies on patterns of word occurrences with Smith Waterman algorithm and Levinstein distance. The extent of data string is altered to another data string is measured by Levenstein distance. These methods are categorized as structured approaches to detect plagiarism in data. The clustering approach is an unsupervised machine learning method that can be prototype based, graph or density based and sometimes they are hybrid algorithms. The ML with Levenstein distance and cosine similarity methods proved to be useful in detecting plagiarism in the digital examinations (Anzén, 2022).
(b) ***Fraud (Malpractice) detection:*** The webcam serves as the human eyes in the virtual environment. The web-based supervision has a webcam as the primary input device for preventing misconduct during the examination time (Hylton, Levy & Dringus, 2016). The fraud detection ML algorithms initially perform the data cleaning and multiple variable creations. The essential features are selected, and the models are trained to detect the incident of potential fraud occurrence (Wei et al., 2020). The generic fraud detection module is given in Fig. 8.

The fraud detection module is designed with ML, and the threshold is set to verify fraud occurrence. The module includes multimodal biometric verification with activity analysis. The detecting module does continuous monitoring, gather data and image to identify any event of fraud in online proctoring (Haytom et al., 2020). In *in-situ* detection, the head pose detection and the gaze tracking with ML is called VFOA (visual focus of attention). This model has a threshold X for cheat detection. When the VFOA value is more significant than X, it alerts the proctor of possible cheating and makes them more vigilant (Indi et al., 2021).

(c) ***Multiple account detection***: In the cases of Massive Open Online Courses (MOOC), many students open numerous fake accounts to obtain a solution for the question. The CAMEO detector based on ML resolves this problem and identifies the master account and the harvester (Ruipérez-Valiente et al., 2019). The correct answer is harvested from any charges to earn a certificate.
(d) **E-cheating intelligence agent:** ML can be incorporated into the examination system as an intelligence agent to detect practices of online cheating. The agent can handle malpractices corresponding to Internet protocol and behavioral changes. The DNN, RNN, and LSTM algorithms are apt to detect such academic dishonesty (Tiong & Lee, 2021).
(e) **Liveliness spoof detection:** The liveliness of the student can be seen for fraud identification. There are cases where the student spoofs the liveliness with photos or images.

The articles with ML spoof identifiers are displayed in Table 3.

**Table 3:** ML techniques in spoof identification.

**Paper ID**	**Spoof identifier**	**ML techniques**
Thepade et al. (2020)	Face	Naïve Bayes, SMV, MLP, Decision table, J48, Random Forest & Random Tree
Patil et al. (2020)	Face	CNN and HAAR cascade classifier
Raj, Tamilselvi & Javith (2022)	Face	3D face with CNN, LBPH, and HAAR cascade classifier
Singh, Joshi & Nandi (2014)	Eye and mouth	Principal component analysis with HAAR cascade
Deepika & Kirithigaa (2021)	Retina movement	Logistic regression with data mining
Nema (2020)	Blink count	Support Vector Machine
Deepika & Philip (2021)	Lip movement	Logistic, Linear, and polynomial regression

(f) **Anomaly detection:** The LSTM algorithm is applied to students’ test scores and compares with their previous scores on assignments, quizzes, and other performance metrics. The anomaly of the student’s final score exceeding the fixed tolerance limit is detected, and the potential cheating or fraud is detected if there are inconsistencies in scores (Kamalov, Sulieman & Santandreu Calonge, 2021).

## ROLE of ML in Post-exams (Evaluation Phase)

### Evaluation and grading

The assessment of online e-learning is part and parcel of the examination system. Machine learning has transfigured the assessment system. The ML with AI provided a breakthrough in the online grading system with automation and more accurate assessment (Gardner, O’Leary & Yuan, 2021). The DT, NB, and SVM are widely used in evaluation techniques to assess students’ learning outcomes. These algorithms can estimate or predict the student’s expected performance (based on their continuous internal assessment) in advance and are used for validity purposes while doing answer script evaluation (Rana et al., 2021). ML has a significant role in adapting to be integrated with other systems to design innovative assessment models. The Item Response Theory with Natural Language Processing is used to build a student assessment method model (Marigowda et al., 2021). The various ML algorithms used in awarding grades are discussed below:

• **Quizzes:** ML predicts responses in advance, enabling the technique to predict and evaluate the exam questions (Lincke et al., 2021). The commonly used algorithms include Linear regression, Logistic regression, gradient boost tree, Bayesian neural network, and XGboostas.
• **Short answers:** The automated short answer grading is possible with classification or regression models—most of the ML algorithms with support vector machines are used extensively for grading short answers online (Galhardi & Brancher, 2018).
• **Essays:** The human manual score for essay evaluation is checked against the ML algorithms such as linear regression, random forest, and support vector machine. The ML algorithms can evaluate essays with automated ML test models and proved to be capable of assessing closer to the manual scores (Ghanta, 2019). The handwritten sheets are converted digitally with optical character recognition and evaluated using the neural networks module for assessment and grading scores (Rosy Salomi Victoria, Viola Grace Vinitha & Sathya, 2020).
• **Content**: When combined with a random forest algorithm, the hierarchical SoftMax algorithm can grade the content bag of words for evaluation. However, the downside of ML with a hierarchical algorithm is that when it is tokenized, the grammatical and vocabulary can be a challenge (Chauhan et al., 2020).
• **Short answers**: The short answers were assessed by KNN, SVM, and Gini are implied, and ML techniques are proved to have the best experience (Çınar et al., 2020).
• **Subjective answers**: The ML processing techniques such as Wordnet and Word movers’ distance (WMD), cosine similarity, and multinomial naïve Bayes (MNB) are employed for assessing the subjective answers; The WMD method proved to be more accurate in automatic evaluation (Bashir et al., 2021).
• **Descriptive answers:** The evaluation of descriptive answers is more challenging due to the evaluation of unstructured data. The faculty answers are stored in the semantic database and is compared with students’ answers. The calculation of semantic similarity is estimated, and the grading is done based on these similarity scores. The comparative semantic analysis of human and ML evaluation was done with TF-IDF (Term Frequency-Inverse Document Frequency), cosine similarity, and LSA. The cosine similarity is closer to the values of manual grading of descriptive answers (Phalke, Bamnote & Ahmad, 2021).

### Issues with ML in examination:

#### Challenges

• The ML-based algorithms do not have a test oracle. These models are sometimes based on probabilistic prediction with no actual values. Though the ML models are crucial for online examination and evaluation, there is a need for random testing with human grading to ensure fairness and reliability of student scores.
• The ML execution consumes ample sample data space for storage and process. There is a need for substantial data spaces for storing colossal student test data supported by higher-end servers. Academic institutions and universities need an update in software architecture, data management, and storage. Updating the storage devices increases the burden of cost-effectiveness for the university or school administration (Marijan, Gotlieb & Kumar Ahuja, 2019).
• The ML decision-making is systematic, and there is a lack of rationale judgment in assessing students’ descriptive answers. On the contrary, human decisions are multifaceted with empathy and flexibility, and reviews are moral or context-related (Webb et al., 2020).
• There are several challenges on the student’s side, such as they should have virtual devices like routers with required internet data coverage in their home network. The students should also possess a laptop or standalone with a webcam and other accessories.
• There is a learning and adaptation process for both students and teachers to the new assessment and exam management system during these lockdown scenarios. These new methods tend to be confusing. The changes require time to adjust and are not liked by both ends. Learning and adapting to change is difficult for students and teachers and consumes time and effort (Bashitialshaaer, Alhendawi & Lassoued, 2021).
• The home environment is supposed to be environment friendly and relaxing to the student’s mentality. When examinations are conducted at home, the students tend to have mental stress because changing from a comfortable and familiar environment to a new hectic environment could cause stress.

### Threats to using ML in the examination:

#### (a) Privacy

Privacy is a big question mark since ML applications require personal or private data for student authentication or verification during the examination. These identity data are stored in a location for comparison and verification before examinations. There is a possibility of hacking these data, such as student addresses, that can be exposed or misused. There is a possibility of privacy issues if these data are mishandled (Al-Rubaie & Chang, 2019). The ML algorithms use private data to design the prediction or evaluation models. These training models are prone to attacks. So, there is a need for privacy in two areas: Training set data privacy and model privacy. The adversaries have two types of access to the training models. The white box access is full access to the training set model with knowledge of the data. On the contrary, black-box access is the access to the prediction model with queries and interference.

In ML, the models for prediction and the private data require assured protection of student data (Liu et al., 2020). Another concern is that the student is monitored on video while writing examinations during an assessment. The General Data Protection Regulation (GDPR) has instructed academic institutions to justify the necessity of video recording. The survey indicates that students’ privacy is violated when closely monitored by their devices such as a webcam. Though that is a need of an hour, the student’s privacy should never be broken during the supervision by monitoring their devices wherever they may be (Langenfeld, 2020).

#### (b) Security

The student data should be ensured private, and privacy always necessitates security against privacy attacks. Privacy and security go hand in hand against threats due to pitfalls in ML. The secure and private data with white box access are attacked less since the attacker has almost all data access on models and private training data. The black box access, the data that are prone to be more secure are targeted for attacks. The ML models should detect such attacks and preserve the model and the privacy of the data (Papernot et al., 2018). The issue with ML is that the assessment models require top-notch security as it contains highly secure data of exam question papers and student data. The disadvantage is that these secure models are cost-heavy. The integrity of these assessment models depends on cost-effectiveness, which may weigh a load on the academic institutions to need more financial support (Langenfeld, 2020). Many attacks or anomalies are induced in the ML system for less security. The attacks are data poisoning, backdoor attacks on the assessment models, interference attacks on sensitive data, and private data stealing. The security methods are inevitable in the ML architecture for security (Xue et al., 2020).

#### (c) Fairness

In the design of online assessment models based on ML, the neural networks and decision-making algorithms are applied in evaluation. These models should judge and evaluate students’ answers with fairness and integrity. The decision trees are like human judgment, but machine algorithms do not assess the students’ tests with human consideration and sensitivity. The ML decision-making algorithm should be ensured with additional information for more fairness (Mehrabi et al., 2021).

### Future solutions of ML

Though ML is vulnerable to attacks, the ML models can detect these breaches and attacks. The ML algorithms can handle authentication, distributed attacks, anomaly, or intrusion detection (Hussain et al., 2020; Liang et al., 2019). With the required additional security features, it can be modified for more security (Kaddoura & Husseiny, 2021; Kaddoura & Al Husseiny, 2021).

#### (a) Privacy-preserving Machine language (PPML)

When the multiple parties train in an educational assessment of the teaching module, the data can be secured privately with cryptographic approaches. The issues and solutions for the ML are tabulated in Table 4.

**Table 4:** Threats and solutions for privacy.

**Threats/attacks**	**Solution**		
Private data in the clear Model inversion Membership De-anonymization	**Cryptographic:** Homomorphic encryption, Garbled circuits, Secret sharing, Secure processors,		**Perturbation approaches:** Differential privacy Local differential privacy Dimensionality reduction
Model extraction Feature estimation Membership interference Model Memorization	**Cryptographic:** Homomorphic Encryption (HE), Cryptonets, Multikey HE	**Obfuscation:** Differential privacy (DP), Simpler DP, DPfed AVG	**Aggregation** Federated learning Private aggregation

**Table 5:** Security attacks and solutions.

**Attacks**	**Solution**
Poisoning attacks	ANTIDOTE, KUAFUDET, AUROR, and Defending SVM
Backdoor attacks	Activated clustering method and STRIP
Adversarial attacks	Fast gradient sign method and Sec defender
Model stealing	ML capsule and PRADA
Sensitive data	PATE
Misuse attacks	VANET, CANN, and KDD
Anomaly attacks	LSTM RNN and RNN IDS
Malware attacks	LSTM

#### (b) Security solutions

Standard security attacks are training set poisoning, backdoor attacks, adversarial example attacks, model theft, and recovery of sensitive training data. The available solutions are provided in Table 5.

## Conclusions

Machine Learning is instrumental in transforming the current field of education under lockdown scenarios. ML forms a basis for machines to learn from real-time experiences and examples. Its learning capability using statistical techniques paves the way for future education. The benefit to students and the potential of the ML to automate learning and assessments is this article’s focus. The lockdown necessitated Machine Learning to transform the education mode to virtual or online. The complete exam cycle from preparation to feedback is the thrust area for lockdown exam management. ML has proved its significance in smart attendance, identifying academically vulnerable students, personalized learning, and predictive analysis of students’ educational performance in the preparation phase before the examination. Automatic scheduling, authentication, proctoring, and quest bank generation are possible with ML algorithms during the examination phase. The feedback on examinations, fraud, and cheat detection and prevention were highlighted for more understanding. ML algorithms also efficiently evaluate all types of questions, be they a quiz or descriptive. This article ends with a note on the threats and challenges such as privacy, security, and fairness. This article is structured comprehensively with an analysis of machine learning methods for Lockdown Exam Management Systems. The future work is planned to bring together the Deep learning concepts and explore the implication of deep learning in exam management systems.

## Additional Information and Declarations

### Data Availability

The following information was supplied regarding data availability: This is a literature review.
